# Involvement of adult children in treatment decision-making for older patients with cancer — a qualitative study of perceptions and experiences of oncology surgeons and nurses

**DOI:** 10.1007/s00520-022-07349-z

**Published:** 2022-09-01

**Authors:** Bea L. Dijkman, Wolter Paans, Hanneke Van der Wal-Huisman, Barbara L. van Leeuwen, Marie Louise Luttik

**Affiliations:** 1 Research Group Nursing Diagnostics, Hanze University of Applied Sciences Groningen, Petrus Driessenstraat 3, P.O. Box 3109 9701 DC, 9714 CA Groningen, The Netherlands; 2 Department of Surgery, University of Groningen, University Medical Center Groningen, P.O. Box 30.001, 9700 RB Groningen, The Netherlands; 3 Department of Critical Care, University of Groningen, University Medical Center Groningen, P.O. Box 30.001, 9700 RB Groningen, The Netherlands

**Keywords:** Older patients, Cancer treatment decision-making, Family involvement, Shared decision-making, Oncology health professionals

## Abstract

**Background:**

Many older patients with cancer have their family members, often their adult children, involved in a process of treatment decision-making. Despite the growing awareness that family members can facilitate a process of shared decision-making, strategies for involving family members are scarce. Furthermore, literature about shared decision-making pays little attention to family involvement or to the impact that family relations have on the decision process. The purpose of this study was to explore how surgeons and nurses perceive the involvement of adult children of older patients with cancer in treatment decision-making. Subsequently, it identified strategies to ensure family involvement in the decision-making process, used in clinical practice.

**Methods:**

Qualitative open in-depth interviews were conducted with 13 surgeons and 13 nurses working in a university or general hospital. Qualitative content analysis was conducted according to the steps of thematic analysis.

**Results:**

Both nurses and surgeons indicated that adult children’s involvement in decision-making about treatment increases when patients become frail. They mentioned several characteristics of adult children’s behaviour during the decision-making process. Most of these characteristics are beneficial, but they also can be challenging. The distinct nature of adult children’s involvement can help older patients with cancer reach better-informed treatment decisions. Health professionals reported six strategies to support positive family involvement in decision-making about treatment.

**Conclusion:**

Adult children may facilitate a process of shared decision-making and help patients reach well-informed treatment decisions. Health professionals’ strategies deliberately support positive family involvement.

## Introduction

The group of older cancer patients for whom surgical treatment is considered can be characterized as heterogeneous in terms of background, diagnosis, and health problems.

Due to increasing multimorbidity and frailty in older patients, these patients face an increased risk of complications and functional decline following surgery, which might seriously impact their quality of life [1–3]. In such complex care situations, a process of shared decision-making (SDM) is preferred to align treatment decisions with what really matters in the ‘every day’ life of the patient [4]. In surgical oncology practice, achieving SDM for older patients is hard to accomplish [3]. These patients might experience health conditions that make it difficult to participate in SDM [5, 6], and their trust in authority might inhibit them from voicing their opinions to a doctor [7].

In general, it is recommended to involve family members in the process of SDM because it is known that partnering with patients and family members contributes to a patient- and family-centred approach [8]. When patients grow older and their frailty increases, they rely on their family members more frequently for practical and emotional support [9–11]. Family members may facilitate SDM, but they can also complicate the SDM process if, for example, they have different views on treatment preferences or on the patient’s capability for involvement in the process [5, 10]. There are indications that challenging situations may arise more often in relationships between patients and their children than between partners [12, 13].

Existing research on SDM has paid little attention to the relationships among patients and their family members or to the impact that these relationships have on the decision-making process [6, 10, 14]. Most research on family involvement in treatment decision-making focuses either on family members in general or on partner involvement in particular. Little is known about the specific role of adult children in the process of treatment decision-making [14, 15].

Surgeons and nurses play a central role in decision-making about the surgical treatment of older patients with cancer [2, 16]. Research examining these professionals’ perspectives provides a deeper understanding about the treatment decision-making process in clinical practice and can help to implement person- and family-based strategies in this process. Therefore, this study explores the perceptions and experiences of surgeons and nurses at the outpatient clinic on the involvement of adult children in treatment decision-making for older patients with cancer. Additionally, it identifies the strategies that surgeons and nurses working in clinical practice use to support the positive involvement of adult children in the treatment decision-making process.

## Methods

### Design and participants

This study was conducted using a qualitative open in-depth interview design according to the consolidated criteria for reporting qualitative research [17]. Purposive sampling was used to select surgeons and nurses who work at oncology outpatient clinics. These participants were recruited from two hospitals in the northern Netherlands: one university hospital and one general centre. The invitation letter was sent via e-mail to 35 participants, including 21 surgeons and 14 nurses.

### Ethical considerations

The ethical committee of the University Medical Center Groningen (UMCG) approved this project’s study protocol (project number: 202000174). All participants were informed about the study’s aim and about their right to withdraw from it. They were asked to sign an informed consent form. Transcripts of the interviews were sent to the participants for approval. All audio recordings and transcripts were stored according to the regulations.

### Data collection

In-depth interviews were used to collect detailed information about participants’ thoughts and behaviours [18]. This study’s five authors developed a guide for open in-depth interviews and revised it during pretesting with one nurse and one oncology surgeon. The open in-depth interviews covered three main topics (box 1). Each topic began with factual questions, then continued to opinion questions. Participants were encouraged to elaborate on topics via open-ended questions such as ‘Can you tell more about…’ and ‘What do you think of…’ [18].

*Box 1: Interview topics*

**Table:** 

a) The involvement of adult children in treatment decision-making for older patients with cancer. Examples from clinical practice.
b) The benefits and challenges of involving adult children in treatment decision-making for older patients with cancer.
c) The practices that current health professionals use to facilitate the positive involvement of adult children in treatment decision-making for older patients with cancer.

The interviews were performed by one investigator, an experienced social scientist (BD). Due to the COVID-19 pandemic, the interviews were conducted either online through video calls over Microsoft Teams or face-to-face in accordance with COVID-19 safety regulations. The recruitment of participants continued until three researchers (BD, ML, and WP) agreed that the interviews presented no new information and that data saturation had been achieved [19]. All interviews were audio recorded and transcribed verbatim.

### Data analysis

Thematic analysis was conducted according to Braun and Clarke [20]. ATLAS.ti 8 software was used to facilitate the process of coding and categorizing. Three researchers (BD, ML, and WP) familiarized themselves with the data, discussed initial coding, and searched for meaningful overarching themes. They discussed different interpretations of the codes and themes until they reached consensus about the final coding framework and the naming of the themes. Reporting the themes included synthesizing the underlying codes. Rigour was addressed via discussions in two weekly meetings attended by three researchers (BD, ML, and WP). These meetings were designed to resolve different interpretations of the data that arose during the analysis process.

## Results

### Participants

In total, 26 of 35 invited health professionals participated in the study with an equal number of surgeons and nurses. The reasons for not participating were a lack of time (*n* = 7) or the failure to meet the inclusion criteria working at an outpatient ward (*n* = 2). Interviews lasted for 20–50 min. Table 1 summarizes the participants’ characteristics.

**Table 1:** Participants’ characteristics

	Surgeons (***n*** = 13)	Nurses (***n*** = 13)
Gender		
Male	10	0
Female	3	13
Years of experience		
Mean (*SD*)	11.5 (7.0)	13.5 (9.9)
Oncology specialization		
Breast & melanoma	1	3
Gastrointestinal & colorectal	10	6
Ear, nose, and throat	2	4

### Qualitative themes

Three main themes emerged from the data: (1) frailty; (2) decision-making process; and (3) health professionals’ strategies.

#### Theme 1: frailty

All surgeons (S1–13) and most nurses (N1–3, 7, 9, 10, 13) reported that, when patients become frail, this can influence their ability to participate in medical conversations. In these situations, adult children’s involvement in medical consultations and treatment decision-making processes increases. Sometimes, adult children even take over the conversation and talk on the patient’s behalf.

“During the medical consultations, some adult children are very interfering, and others are very much in the background. I think it has a lot to do with whether they feel like their father or mother understands it themselves. (S1)”

When the patient’s partner is frail, or when the patient has no partner, adult children become more actively involved to support their parent in the treatment decision-making process (S1, 4, 7, 8; N2, 5, 13). Participants explained that most older patients with cancer consider their partner to be their closest family member.

“Among patients younger than 85, the partner is often the most important. Above that, it’s mostly the children who are. That is because the partner has either passed away or the partner has physical and/or mental issues. (S8)”

#### Theme 2: decision-making process

**Fig. 1:**
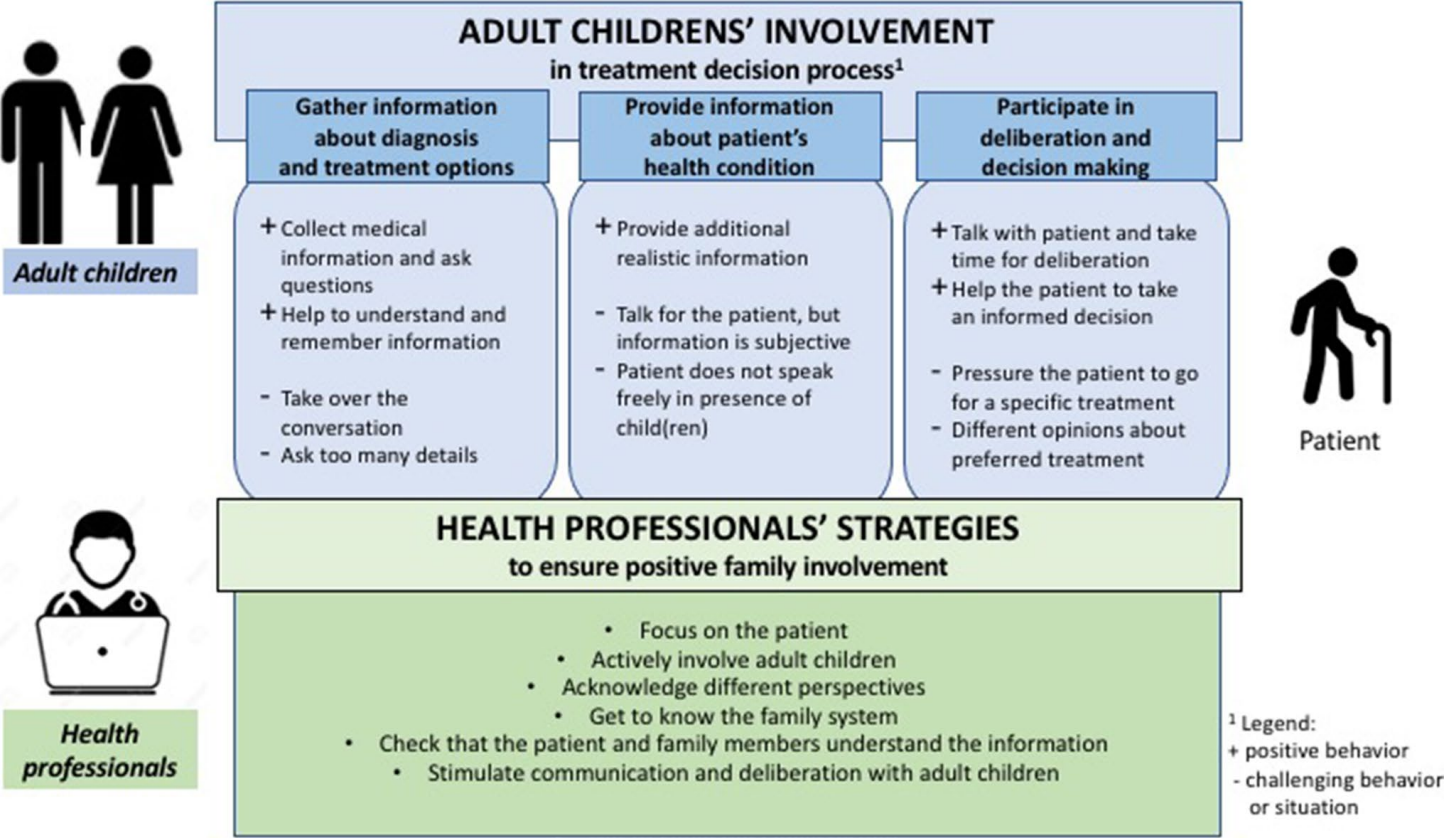
Health professionals’ perceptions of adult children’s involvement in triadic treatment decision-making processes for older patients with cancer and these professionals’ strategies to ensure positive involvement

The data revealed characteristics of adult children’s involvement in three steps of the decision-making process (see Fig. 1).

## Gather information about treatment options

Adult children support the older patient by collecting, understanding, and recalling information about the diagnosis and treatment options (S1–8, 10–13; N1, 2, 6, 8, 10–12). Adult children more often than partners prepare questions and collect information beforehand through the Internet. During medical consultations, adult children focus on medical information and tend to ask more questions than patients themselves.

“... they are much more critical and ask also questions about the disease itself. (N11)”

It becomes challenging for surgeons and nurses when children request too many details that do not interest the patient (S4, 8; N1, 12).

## Provide information about the patient’s health condition

The information that adult children provide about the patient’s health conditions and daily functioning is considered to be valuable (S1–10, 12, 13; N1–6, 9, 10, 12, 13). Adult children promote their opinions when they think that some important piece of information is missing. Participants noticed that, in many cases, this information about the patient’s health condition is more realistic than the information that the patient or partner provides. To explain this, participants suggested that patients and partners are used to the gradual decline of their abilities or that they do not totally accept the situation in which they experience difficulties performing their daily activities.

“… it is an extra bit of information that the patient does not give out. When someone, for example, has cognitive issues and says something like, “I am not suffering from anything,” but you notice throughout the conversation that some memory issues are possible, then they can comment, like, “in the home situation I notice this or that”… (N3)”

Challenging situations arise when the patient withholds personal information in the presence of children (S6; N3, 12) or when adult children provide subjective information directed towards their personal treatment preferences (S2, 4; N10).

## Participate in the deliberation process and the treatment decision

When adult children are involved in the deliberation process, patients seem to spend more time discussing the diagnosis, the different treatment options, and the impact that these options might have on daily life. This discussion is considered to be very positive because it helps the patient make a better-informed treatment decision (S3–9; N1–3, 5, 7–11).

“... it is nice if family is involved, of course, because there are aspects that the patient does not immediately take in, but can later discuss with his children ... And then, in the end, the same choice for an operation might be made, but involvement of children influences whether or not the decision was deliberately made. (S5)”

Challenging situations arise when adult children bring their own treatment preferences and put emotional pressure onto the patient (S1, 3, 4, 6, 7, 9, 11; N2–8, 10, 11, 13). More often than partners, adult children may prefer that the patient undergo intensive treatment to lengthen their life. Partners are more likely to agree with the patient’s treatment preference and to empower the patient to make their own decision.

“The partner tends to go more with what the patient wants, and the children can sometimes question that and want something different or want it to be more extensive. (N7)”

### Theme 3: health professionals’ strategies

The data revealed six strategies that both surgeons and nurses use to stimulate the positive involvement of adult children in the decision-making process of treating older patients with cancer (Fig. 1). The different roles of surgeons and nurses are reflected in the way that these strategies are used in clinical practice.

## Focus on the patient

Participants raised the importance of always keeping the focus on the patient throughout the treatment decision process (S1, 4–9, 11, 12; N1–4, 6–8, 10, 12). It is key to ask the patient directly, as illustrated in the quote below:

“I always ask the patient himself: “What do you really want? How do you feel about it?” (S7)”

This strategy is useful in situations when adult children dominate the conversation, and the patient takes a passive role as well as in situations when different parties express conflicting treatment preferences. Some surgeons stated that they might refer to the treatment agreement (S3, 9, 11–13).

“...I have a treatment agreement with the patient and not with the family. So, in the end, we do what the patient wants, and not the family. I clarify this, if I need to. (S13)”

Four nurses (N2, 7, 8, 12) saw it as part of their role to emphasize that the patient has a choice and that the patient is the one who needs to agree fully with the chosen treatment.

## Acknowledge different perspectives

Several surgeons and nurses considered it important to acknowledge the different opinions and emotions that patients, their partners, and their adult children might have (S1, 3, 11; N6–8, 10, 12, 13). Surgeons and nurses mentioned their strategy of identifying what they hear or see.

“… if I feel like the conclusion is made by the children and not by the patient. Well then, I mention that: “I hear you, and I understand you and I will come back to that in a little while.” Then I look the patient right in the eyes, sit right in front of him and say, “I really want to hear it from you.” (S11)”

Participants stated that this strategy helps patients and their family members feel heard and understood. Adult children can be more supportive towards patients during the deliberation process if they better understand the different perspectives.

## Involve adult children

Two surgeons and two nurses (S10, 11; N9, 12) mentioned that it is important to actively involve adult children throughout the treatment decision process. This includes welcoming the family members, inviting them to ask questions, and asking their opinion about patient’s health condition and situational circumstances.

“Try not to focus only on the patient, but also involve family members. Ask what they prefer and say that they can ask questions as well if they don’t understand it. (N9)”

A few participants mentioned that actively involving adult children in the treatment decision process has a positive effect on the care trajectory and on their role as caregivers.

“So, involve them in this to make sure that it succeeds for everyone. For the patient, for the family and also in connection to the hospital, the care relationship. (N12)”

## Get to know the family system

Some participants (S8; N4, 9–11) explicitly mentioned the importance of getting to know the family system by asking questions about the family and the patient’s support network. When the patient is accompanied by one or more family members, health professionals observe the interactions and attempt to make a first impression about family functioning and relationships (S3–5, 9, 11–13; N1, 3, 5, 7–9, 11, 12).

“... and I ask also “How is the relationship with the children?” and “How are they involved?” You try to get a sense of that. (S8)”

In some families, relationships may be stressful or conflicted, and communication problems may exist. Health professionals are careful to address this, and, in exceptional cases, they contact the general practitioner or another expert (S6, 9; N1, 6, 10, 11, 13).

## Check that the patient and family members understand the information

Participants mentioned the importance of checking that the patient and family members understand the information provided during medical consultations. Surgeons emphasized that they need to explain a lot of medical information clearly, often in a limited amount of time (S2–4, 6, 7, 9–12). After the consultation with the surgeon, nurses have additional time to speak with the patient and the family members. During this time, they check whether everyone understands the information and the consequences that the treatment options will have on daily life (N1–13). Some explained that this procedure is of foremost importance when there are different treatment preferences between patients and their adult children. When the children realize the risks and the impact on the patient’s daily life, this might dissolve any disagreement about the decision and encourage them to better support the patient in the deliberation process.

“Then I ask the patient questions: “Do you know what you want?” and “How far do you want to go?” ... “Do you accept to lose your independence?” ... Then you see that children start to realise what the consequences are. That will change the further conversation... (N7)”

## Stimulate communication and deliberation with adult children

The data revealed three ways that participants stimulate communication and deliberation between patients and their adult children at home (S3, 6, 7, 11; N1–13). First, they address sensitive topics — such as grief, death, loss of independence, and caregiver burden — during the medical consultations. This makes it easier for patients and their children to continue speaking about this at home. Second, they advise patients and their children to talk about what is important for the patient in daily life.

“So, in case there are family members accompanying the patient, then I know they will talk about it later, and I can give them some homework. It might be too much information at once, and it is a big decision. Then I suggest, “Don’t try to answer all questions, but focus on only one or two questions. For example, how would it be for you if that would happen?” And we will talk about it next time. (S11)”

Third, nurses and surgeons encourage patients and family members to talk with the general practitioner about what decision to make (S7, 9; N10).

## Discussion

This study investigated how surgeons and nurses in clinical practice perceive the distinct nature of adult children’s involvement in decision-making about the treatment of older patients with cancer. In line with other studies, both surgeons and nurses confirmed that the role of adult children in decision-making about treatment increases when patients and their partners become frail and when, for example, their cognitive abilities decline [10, 11, 21].

Surgeons and nurses valued the specific role of adult children in the treatment decision-making process. They described adult children as being proactive in gathering information, less reluctant than partners to ask questions, and supportive when it came to providing information about the patient’s health condition. Adult children can stimulate deliberation and move the conversation beyond a mere medical perspective by considering relevant aspects of a patient’s life. Nevertheless, challenging situations arose as well when patients withheld information in the presence of their children; when adult children dominated conversations; or when children had a different treatment preference — often, one that was more life-prolonging — from the patient. Our results surpass those of previous studies [10, 13, 14], by showing that the aforementioned benefits and challenges seem to occur more often in relationships between older patients and their adult children than in relationships between partners.

The surgeons and nurses in our study mentioned several intuitive strategies that they practice to ensure the positive involvement of adult children in decision-making about treatment (Fig. 1). The first strategy that participants mentioned reflects the traditional way of looking at SDM: ‘always keep the focus on the patient’. In relation to this strategy, participants referred to the treatment agreement between physicians and patients as the basis for the SDM process. At the same time, participants recognized that positive family involvement presupposes good family relationships and communication skills. These factors were underpinned by the strategies ‘get to know the family system’ and ‘acknowledge different perspectives’. Although the roles of physicians and nurses were not a focus of the current study, interviews showed that they seem to differ from each other. This aligns with other studies’ findings, which described these professions’ complementary roles in the cancer treatment decision-making process [16, 22].

Several studies have advocated a more patient- and family-focused approach in decision-making about cancer treatment, but the bulk of the scientific literature on SDM still focuses on the physician–patient dyad [6, 10, 23]. As described by our participants, everyday practice seems to call for a paradigm shift towards a patient- and family-centred approach in SDM. Involving family members in difficult treatment decisions requires practitioners to possess specific knowledge of and competencies in fields such as family dynamics and triadic conversation skills. The existing literature on treatment decision-making often lacks practical tools or strategies for how to involve family members [23, 24]. It is a major challenge for healthcare professionals to achieve high-quality and balanced treatment decisions in families whose mutual relationships and communication skills are not always optimal. Communication strategies, as developed by Laidsaar-Powell et al. [25, 26], can help oncology physicians and nurses better communicate with, and support, caregivers during decision process. Additionally, the core elements of a family systems approach, combined with family health conversations (FHCs) regarding treatment decision-making, could be helpful in developing tools and strategies to improve shared treatment decision-making for older patients with cancer in clinical practice. As developed by nurse scientists, the typical components of FHCs include getting to know the family, acknowledging different perspectives, and stimulating communication within the family [27, 28]. Although conversations about treatment decision-making serve a specific goal, the components and theoretical underpinnings of FHCs could help healthcare workers develop practical strategies for triadic conversations related to treatment decision-making.

The fact that both surgeons and nurses were interviewed about how they perceive and interpret their roles provided a nuanced picture of the conversations, challenges, and applied strategies that are involved in cancer treatment. Because this study reached data saturation, its findings are assumed to provide a reliable and valid answer to the research questions. Due to the small number of participants, however, the findings should be treated cautiously; they are not necessarily transferable to other settings or cultures.

## Conclusion

The perceptions and experiences of surgeons and nurses revealed specific characteristics of adult’s children involvement in decision-making about the treatment of older patients with cancer. Surgeons and nurses perceive most of these characteristics as beneficial. According to surgeons and nurses, adult children seem to facilitate a process of SDM for older patients with cancer and to help these patients reach well-informed treatment decisions. Therefore, surgeons and nurses stimulate the communication and deliberation between these patients and their adult children. However, involving family in treatment decision-making also triggers specific complexities and challenges in treatment decision conversations that seem to call for the development and implementation of practical patient- and family-centred strategies.

## Data Availability

The data that support the findings of this study are available on request from the corresponding author.
